# Oral colonisation by antimicrobial-resistant Gram-negative bacteria among long-term care facility residents: prevalence, risk factors, and molecular epidemiology

**DOI:** 10.1186/s13756-020-0705-1

**Published:** 2020-03-04

**Authors:** Mi Nguyen-Tra Le, Shizuo Kayama, Mineka Yoshikawa, Toshinori Hara, Seiya Kashiyama, Junzo Hisatsune, Keiko Tsuruda, Makoto Onodera, Hiroki Ohge, Kazuhiro Tsuga, Motoyuki Sugai

**Affiliations:** 1 Project Research Centre for Nosocomial Infectious Diseases, Hiroshima University, Hiroshima, Japan; 2 Department of Antimicrobial Resistance, Hiroshima University Graduate School of Biomedical & Health Sciences, Hiroshima, Japan; 3 Antimicrobial Resistance Research Centre, National Institute of Infectious Diseases, Higashi Murayama, Japan; 4 Department of Advanced Prosthodontics, Hiroshima University Graduate School of Biomedical and Health Sciences, Hiroshima, Japan; 5 Clinical Laboratory, Hiroshima University Hospital, Hiroshima, Japan; 6 Department of Oral Epidemiology, Hiroshima University Graduate School of Biomedical & Health Sciences, Hhiroshima, Japan; 7 Department of Infectious Diseases, Hiroshima University Hospital, Hiroshima, Japan

**Keywords:** Antimicrobial resistant bacteria, Oral colonisation, Extended-spectrum ß-lactamases, Carbapenemase, *Escherichia coli*, *Acinetobacter baumannii*, *Acinetobacter ursingii*, *Pseudomonas aeruginosa*, Risk factor analysis

## Abstract

**Background:**

For residents of long-term care facilities (LTCFs), antimicrobial-resistant bacteria (ARB) are a risk factor, yet their oral colonisation, potentially leading to aspiration pneumonia, remains unclear. This study was undertaken to survey the prevalence, phenotypic characteristics, and molecular epidemiology of antimicrobial-resistant Gram-negative bacteria in the oral cavity of LTCF residents, and to analyse the risk factors for such carriers.

**Methods:**

This study involved 98 residents of a LTCF in Hiroshima City, Japan, aged between 55 and 101 years. Oropharyngeal swabs were collected and plated on screening media for ESBL-producing and carbapenem-resistant bacteria; isolates were identified and tested for antibiotic susceptibility; biofilm formation was tested in vitro; identification of epidemic clones were pre-determined by PCR; resistance genes, sequence types, and whole-genome comparison of strains were conducted using draft genome sequences. Demographic data and clinical characterisations were collected and risk factors analysed.

**Results:**

Fifty-four strains from 38% of the residents grew on screening media and comprised predominantly of *Acinetobacter* spp. (35%), *Enterobacteriaceae* spp. (22%), and *Pseudomonas* spp. (19%). All *Escherichia coli* isolates carried CTX-M-9 group and belonged to the phylogroup B2, O25:H4 ST131 *fimH30* lineage. Six *Acinetobacter baumannii* isolates presented identical molecular characteristics and revealed more biofilm production than the others, strongly suggesting their clonal lineage. One *Acinetobacter ursingii* isolate displayed extensive resistance to various ß-lactams due to multiple acquired resistance genes. One *Pseudomonas aeruginosa* isolate showed exceptional resistance to all ß-lactams including carbapenems, aminoglycosides, and a new quinolone, showing a multidrug-resistant *Pseudomonas aeruginosa* (MDRP) phenotype and remarkable biofilm formation. Genome sequence analysis revealed this isolate was the *bla*_IMP-1_-positive clone ST235 in Japan. Strokes (cerebral infarction or cerebral haemorrhage) and percutaneous endoscopic gastrostomy tubes were recognised as risk factors for oral colonisation by ARB in the LTCF residents.

**Conclusions:**

ARB, as defined by growth on screening agar plates, which carried mobile resistance genes or elements or conferred high biofilm formation, were already prevalent in the oral cavity of LTCF residents. Health-care workers involved in oral care should be aware of antimicrobial resistance and pay special attention to transmission prevention and infection control measures to diminish ARB or mobile resistance elements dissemination in LTCFs.

## Background

Extensive consumption of antibiotics has been the crucial pressure impelling drug-resistance, leading to the global concern of multi-antimicrobial resistant microorganisms. While much attention has been paid to infectious diseases by multidrug-resistant (MDR) organisms in severely ill patients in intensive care units, the colonisation by antimicrobial-resistant bacteria (ARB) among patients in long-term care facilities (LTCFs) has been thus far neglected. Since the early 1970s, various reports have repeatedly emphasised the role of asymptomatic patients in LTCFs as a reservoir of ARB thus favouring their persistent colonisation and extensive dissemination throughout hospital settings [1].

A similar situation is developing across Japan, where the percentage of elderly over 65 years is predicted to be as much as 38.1% [2] by 2060, whilst health care-associated infection management in LTCFs is straggling in comparison with the one in medical settings. Adequate active surveillances still have to clarify the colonisation rates by MDR bacteria among the elderly residing in LTCFs nationwide [3]. Moreover, surveillances for ARB carriage among LTCFs residents have been almost exclusively for faecal or rectal samples [4].

The oral cavity is constantly exposed to external material during its function of air, beverage, and food intake, thus frequently exposed to various microorganisms. Specific histological features of the mouth (e.g., the papillary dorsal tongue, the mucosal epithelia of cheeks, the teeth enamel, and the periodontal surfaces), in addition to changes in the ecological condition (e.g., the salivary flow) may result in the accumulation of host-produced extracellular pellicles and the subsequent formation of a biofilm, a favourable matrix for nutrition supply. Exposure of bacteria to such pellicle-facilitated surfaces over several hours promotes the colonisation by those pathogens in this biotic niche [5]. The persistence of such organisms in the oropharyngeal area, in turn, places the patients at risk for bacterial pneumonia [6].

While previous published data of this target population mainly focused on faecal or rectal samples to evaluate the carriage of ARB [4], a survey by March A. et al. indicated a proportion of ARB present in the oropharyngeal area in addition to the other sites [7]. However, to date, few researchers have addressed this issue in the oral cavity. For those reasons, this paper addresses (i) the prevalence of oral colonisation by ARB, including extended-spectrum ß-lactamases (ESBLs)-producing and carbapenem-resistant bacteria, among the LTCF residents, (ii) risk factors associated with colonisation by AMR organisms, and (iii) molecular epidemiology to figure out if any further necessary preventive measures should be implemented.

## Materials and methods

### Bacterial strains

*Pseudomonas aeruginosa* PA160071 and PA058447, strong biofilm-forming strains, and *P. aeruginosa* PAO1 served as controls. PA160071 is a *bla*_IMP-1_-positive ST235 In113-carrying *P. aeruginosa* (carrier of type E integron [8])*,* which is similar to NCGM2.S1, an epidemic MDR ST235 *P. aeruginosa* isolated in North Japan (Tohoku region) [9, 10]. PA058447 is a *bla*_IMP-1_-positive type F integron-carrying MDR *P. aeruginosa*, an epidemic ST235 in the Hiroshima region [11].

### Study design and participants

This study was performed to assess the prevalence of oral and pharyngeal carriage of antimicrobial-resistant Gram-negative bacteria in an LTCF, from February 2017 to January 2018. A total of 98 residents in a geriatric facility located in Hiroshima city, Hiroshima Prefecture, were included in the study. Informed consent was obtained from the residents or their relatives. This study has been approved by the ethical committee of the Hiroshima University Hospital review board (E-1704).

### Phenotypic characterisation

To screen for the carriage of ARB, oropharyngeal swabs were taken from all patients and spread directly onto CHROMagar™ ESBL and CHROMagar™ mSuperCARBA™ medium plates (Kanto Chemical, Japan). CHROMagar™ ESBL is a screening medium for rapid and presumptive identification of ESBL-producing *Enterobacteriaceae* [12]. Although the formulation of this medium is proprietary, the CHROMagar™ ESBL supplement allows the detection of ESBL-producing bacteria while inhibiting the growth of other bacteria, including most of those carrying *ampC* type resistance. CHROMagar™ mSuperCARBA™ is a screening medium for the detection of carbapenemase-producing *Enterobacteriaceae* [13]. Plates were incubated for 18–24 h at 37 °C and colony growth observed after incubation. All positive colonies were sub-cultured onto secondary plates to confirm the resistance, and those grown on Candida GE plate were eliminated. Bacteria grown on the second screen were considered ARB.

Identification and antimicrobial susceptibility tests were conducted using Vitek-2 System (bioMérieux, France). Species unidentifiable by this system were further investigated using 16S rDNA sequencing and MALDI-TOF MS-Biotyper (Bruker Daltonics, Yokohama, Japan). The antibiotics used for the susceptibility tests included: Ampicillin, Piperacillin, Tazobactam/Piperacillin, Ampicillin/Sulbactam, Cefazolin, Ceftazidime, Cefotaxim, Cefozopran, Cefoperazone/Sulbactam, Cefpodoxime, Imipenem, Meropenem, Doripenem, Amikacin, Ciprofloxacin, Levofloxacin, Minocyclin, Fosfomycin, Sulfamethoxaxole, and Azidothymidine. Results were interpreted according to guideline M100-S25 from the Clinical Laboratory Standards Institute (CLSI) [14].

### Biofilm formation assay

To evaluate biofilm formation of the strains, a biofilm assay using polystyrene plates was conducted as described previously [15], with a few modifications. Each isolate was cultured in 5 mL of Luria-Bertani (LB) broth and incubated overnight at 37 °C. The cultures were diluted 100 times in LB and 10 μL aliquots were transferred to 96-well flat bottom polystyrene plates (Nippon Genetics Co., Japan) containing 100 μL LB or LB plus 1% glucose. Thereafter, plates were incubated at 37 °C for 24 h. Further, plates were gently washed three times with 300 μL phosphate buffered saline (PBS) followed by staining with 1% crystal violet for 15 min. Afterwards, the plates were washed by immersion in a water tub and stirred 10 times to eliminate the unbound crystal violet. The stained biofilms were solubilised in 200 μL of 33% glacial acetic acid for 15 min followed with a 10-fold dilution; OD_590 nm_ was determined with the Varioskan® Flash spectral scanning multimode reader (Thermo Fisher Scientific, USA).

### Genomic characterisation

To check for the presence of resistance elements among the isolates, PCR amplification was performed to screen the extended spectrum ß-lactamase genes group (*bla*_CTX-M-1_, *bla*_CTX-M-2_, *bla*_CTX-M-8_, *bla*_CTX-M-9_, *bla*_TEM_ and *bla*_SHV_) and carbapenemases genes (*bla*_IMP_, *bla*_OXA_, *bla*_NDM_, *bla*_KPC_, *bla*_VIM_ and *bla*_FIM_) (Supplementary Table 1) using Quick Taq HS DyeMix (Toyobo, Japan). The cycling protocol was as follows: denaturation at 95 °C for 5 min, 35 cycles of 95 °C for 30 s, 52 °C for 30 s, 68 °C for 90 s, and 72 °C for 5 min.

We further selected the organisms of interest from those having higher prevalence across our study, namely *Acincetobacter* spp., *Pseudomonas* spp., and *Escherichia coli.* For these isolates, the clonal identification and molecular typing was carried out with the PCR-based ORF Typing method (POT method) [16] using specific POT kits: Cica Geneus® Acineto POT KIT, Cica Geneus® *E. coli* POT KIT, and Cica Geneus® Pseudo POT KIT (Kanto Chemical, Japan) for each strain according to the manufacturer’s instructions. The POT codes were converted from the results of electrophoretic band patterns. The isolate characterisation (epidemic clones, sequence type, resistance genes) and homology between strains were subsequently interpreted.

To further determine the phylogenetic group (A, B1, B2, and D) of *E. coli* strains, a rapid and simple protocol was performed as described previously [17]. In brief, three pairs of primers were used: ChuA.1 and ChuA.2, YjaA.1 and Yja.2, and TspE4C2.1 and TspE4C2.2 (Supplementary Table 1) to amplify the DNA fragments of 279, 211, and 152 base pairs, respectively. The thermal cycle conditions were as follow: denaturation for 4 min at 94 °C, 30 cycles of 30 s, 94 °C; 30 s, 65 °C; 30 s, 72 °C; followed by 5 min at 72 °C. The clades of *E. coli* sequence type (ST) 131 were identified according to Matsumura et al. [18].

The isolates, which were high biofilm producers, resistant to multiple antimicrobials by MICs or positive for antimicrobial-resistance (AMR) genes by PCR, were selected for genome sequencing. Whole genome sequences (WGS) of *E. coli* isolates 43E, 73E, 77E, and 95E, *A. baumannii* isolates 13C, 41C, 45C, 50E-B, 51E, and 52E, *A. ursingii* isolate 56C, and *P. aeruginosa* isolate 71E were obtained using Illumina MiSeq sequencing platform, followed by annotation with the Rapid Annotation using Subsystem Technology (RAST) version 2.0 [19]. The multilocus sequence typing (MLST) and acquired AMR genes were identified with the MLST and ResFinder pipeline [20, 21] available from the Center for Genomic Epidemiology (Lyngby, Denmark) with default settings and whole genome sequence (WGS) raw reads for the analysis. Genome comparison of *E. coli* isolates 43E, 73E, 77E, and 95E was performed using Blast Ring Image Generator (BRIG) version 0.95 [22] with reference to the complete WGS of *E. coli* K-12 MG1655 strain (accession number NC_000913), a well-studied and approximated wild-type *E. coli*, and two ST131 *E. coli* namely *E. coli* EC958 (HG941718) and *E. coli* H105 (CP021454) strains. Similarly, genome comparison of *A. baumannii* 13C, 41C, 45C, 50E-B, 51E, 52E, and 56C isolates was performed with reference to the complete WGS of *A. baumannii* ATCC17978 strain (NZ_CP018664), and genome comparison of *P. aeruginosa* PA71E and PA058447 strains was performed with reference to the available complete WGS of NCGM2.S1 (AP012280) and PAO1 (NC_002516) strains.

### Epidemiological investigation and data analysis

To investigate the risk factors for colonisation by ARB, demographic data of the patients were retrieved from their hospital records, including age, sex, LTCF ward, comorbidities (dementia, strokes (cerebral infarction or cerebral haemorrhage), cardiovascular diseases, diabetes, cancers, fractures, arthropathy), oral motor dysfunction (dysarthria or dysphagia), oral care ability, and denture wearing.

Univariate statistical method (*X*^*2*^ test for categorical variables) was performed to compare the baseline demographics and clinical variables between the patients who were colonised with ARB and those who were not. Associations were analysed using chi-square test or Fisher’s exact test. Odds ratios (OR) and 95% confidence interval (CI) were displayed to indicate the relation strength. All analyses were performed using IBM SPSS statistics software, version 22. Statistical significance was designated as a two-sided *P* value ≤ 0.05.

## Results

### Epidemiological investigation and risk factors associated with colonisation

Overall, 98 patients from the LTCF were recruited and participated in this study. The mean age was 83.34 ± 9.47 years (55 to 101 years old), 39 were men (30%) with a mean age of 80.76 ± 9.44 years (60 to 96 years old) and 69 women (70%) with 84.42 ± 9.34 years (55 to 101 years old).

**Fig. 1:**
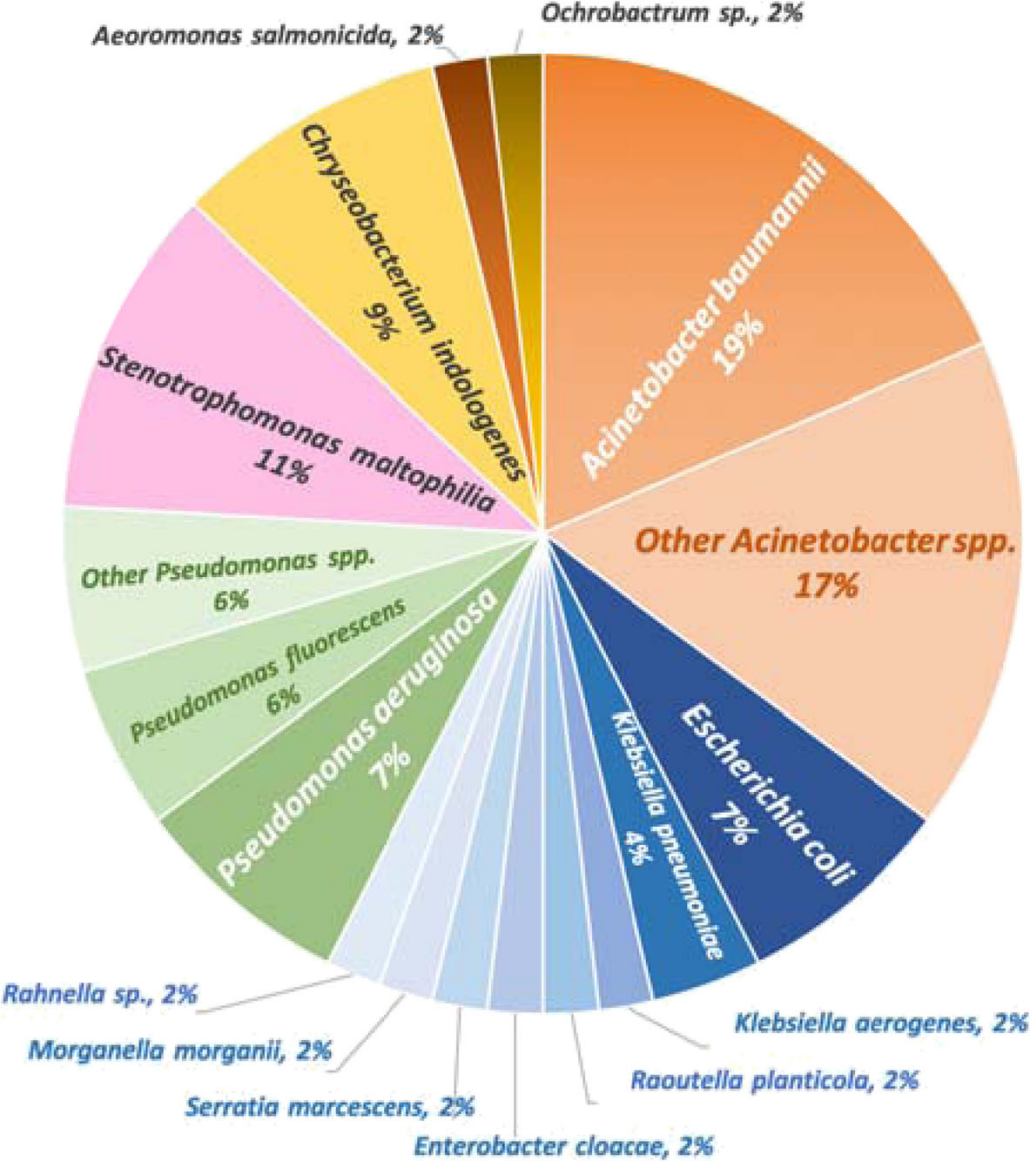
The percentage of antimicrobial-resistant bacteria isolated from oral pharyngeal samples

From the screening by screening media, 51 ARBs were detected by CHROMagar™ ESBL and 49 ARBs were detected by CHROMagar™ mSuperCARBA™. Altogether, a total of 54 isolates from 37 patients (38%) were detected as ARB using screening agar plates. Those isolates belonged to three main genera: *Acinetobacter, Enterobacteriaceae,* and *Pseudomonas* spp*.* (Fig. 1)*.* The number of isolates for each species is listed in Supplementary Table 2. The most common species comprised *A. baumannii* (10 isolates, 19%), *Stenotrophomonas maltophilia* (6 isolates, 11%), *Chryseobacterium indologenes* (5 isolates, 9%), and *E. coli* (4 isolates, 7%). Of 98 residents enrolled, 27% were colonised with a single ARB, 7% were colonised with two ARBs, and 4% were colonised with three ARBs. Among these 54 ARBs, 46 isolates from 32 patients (33%) were confirmed as having reduced susceptibility to one or more tested antimicrobial agents, 29 isolates from 23 patients (23%) were confirmed as cephalosporin-resistant ARB, and 13 isolates from 13 patients (13%) were confirmed as carbapenem-resistant ARB by Vitek-2.

Demographics and clinical characteristics of all patients divided in two groups, ARB-positive and ARB-negative, are shown in Table 1. Two risk factors, strokes (cerebral infarction or cerebral haemorrhage) (OR 3.46, 95% CI 1.38–8.70, *p* = 0.007) and percutaneous endoscopic gastrotomy tubes (PEG tubes) (Fisher’s exact test, *p* = 0.002), indicated the existence of ARB in the oral cavity. In particular, 43.2% (*n* = 16) of ARB-positive individuals underwent strokes, outweighing the percentage of ARB-negative individuals (18% (*n* = 11)). Similarly, 16.2% (*n* = 6) of the residents who were ARB-positive carried PEG tubes, exceeding the percentage of the group of ARB-negative residents (0.0% (*n* = 0)). To a lesser extent, the percentage of patients with dysarthria or dysphagia, diabetes, denture wearing, and male sex tended to be much higher in the ARB-positive group than in the ARB-negative group though no statistically significant differences were found.

**Table 1:** Clinical characteristics of patients and risk factors associated with oral colonisation by resistant bacteria

Variable	Subjects with ARB-positive, *n* = 37	Subjects with ARB-negative, *n* = 61	Univariate analysis		*P*-value
	n (%)		OR	95% CI	
Age ≥ 90 years, n (%)	12 (32.4)	13 (21.3)	1.77	0.71–4.45	0.22
Male sex, n (%)	14 (37.8)	15 (24.6)	1.87	0.77–4.52	0.16
Ward, n (%)					0.92
1^st^ floor	10 (27.0)	17 (27.9)	0.96	0.38–2.40	0.93
2^nd^ floor	14 (37.8)	25 (41.0)	0.88	0.38–2.03	0.76
3^rd^ floor	13 (35.1)	19 (31.1)	1.20	0.50–2.85	0.68
Dementia	15 (40.5)	31 (50.8)	0.66	0.29–1.51	0.32
Strokes (cerebral infarction/ cerebral haemorrhage)	16 (43.2)	11 (18.0)	3.46	1.38–8.70	0.007^**^
Cardiovascular diseases	15 (40.5)	19 (31.1)	1.51	0.64–3.53	0.34
Diabetes	10 (27.0)	10 (16.4)	1.89	0.70–5.10	0.21
Cancers	1 (2.7)	5 (8.2)			0.40^a^
Fractures	5 (13.5)	11 (18.0)	0.71	0.23–2.24	0.56
Arthropathy	2 (5.4)	2 (3.3)			0.63^a^
Dysarthria or dysphagia	13 (35.1)	12 (19.7)	2.21	0.88–5.57	0.09
Inappropriate oral care	18 (48.6)	26 (42.6)	1.28	0.56–2.90	0.56
Denture wearing	24 (64.9)	32 (52.5)	1.67	0.72–3.88	0.23
Percutaneous endoscopic gastrotomy tubes (PEG tubes)	6 (16.2)	0 (0.0)			0.002^a,**^

*OR* Odds ratio, *CI* Confidence interval

^a^ Fisher’s Exact Test

*P value ≤ 0.05, **P value ≤ 0.01

### Phenotypic and genomic characterisation

The presence of PCR-based POT code and antibiotic susceptibility of individual strains through Vitek-2 are presented in Tables 2. All four *E. coli* isolates were resistant to many ß-lactams and ciprofloxacin. Phylogenetic analysis indicated their phylogroups as B2 lineage: positive for all *chuA* genes, *yjaA* genes, and the TSPE4C2 DNA fragment. The POT method identified all those isolates belonging to ST131 carrying *bla*_CTX-M-9_ group gene. ST131 clade PCR assay and C1-M27 subclade PCR assay [18] revealed all those isolates pertained to the C1-M27 subclade. Furthermore, this implied the O serotypes and *fimH* types of O25b-*fimH30* and the presence of *bla*_CTX-M-27_, a subgroup of CTX-M-9 group. WGS of these isolates confirmed their ST131, serotype O25:H4, *fimH30*, and carried multiple plasmid-mediated AMR genes (Table 3). In terms of ß-lactam resistance genes, *E. coli* isolates 43E, 73E, and 77E were found to carry *bla*_CTX-M-27_, while *E. coli* isolate 95E carried *bla*_CTX-M-14_ (belonged to CTX-M-9 group) and *bla*_TEM-1B_. Besides, their DNA sequences of the chromosomal QRDRs (quinolone resistance-determining regions) of *gyrA* and *parC* showed 100% identity with those of MDR ST131 *E. coli* EC958 and H105 strains, which conferred point mutations involving amino acid substitutions. Substitutions were identified at two codons of GyrA: 83 (Ser → Leu) and 87 (Asp → Asn), and two codons of ParC: 80 (Ser → Ile) and 84 (Glu → Val). Genome comparison of these *E. coli* isolates with two MDR ST131 *E. coli* strains (EC958 and H105) through BRIG using the complete WGS of *E. coli* K-12 MG1655 as a reference, revealed high homology among our four isolates and EC958 and H105 strains with identical missing fragments in comparison with K-12 MG1655 (Fig. 3a), suggesting their close origin.

**Table 2:** Genotypic characterisations by PCR and antimicrobial susceptibility of all ARB isolates

Isolate’s code	Species	Patients’ ward (floor)	POT code	MIC (μg/mL)														
				ABPC	PIPC	T/P	A/S	CEZ	CAZ	CTX	CZOP	CPDX	IMP	MEPM	AMK	CPFX	FOM	ST
61E	*Acinetobacter baumannii*	3	8–13-0	–	4 (S)	≤8 (S)	≤2 (S)	–	1 (S)	4 (S)	≤0.5 (S)	–	1 (S)	≤0.25 (S)	≤1 (S)	≤0.25 (S)	> 128 (R)	≤20 (S)
66C	*Acinetobacter baumannii*	3	8–13-0	–	16 (S)	≤8 (S)	≤2 (S)	–	2 (S)	8 (S)	≤0.5 (S)	–	≤0.25 (S)	≤0.25 (S)	≤1 (S)	≤0.25 (S)	> 128 (R)	≤20 (S)
25E	*Acinetobacter baumannii*	2	32–20-41	–	16 (S)	≤8 (S)	> 16 (R)	–	4 (S)	16 (I)	> 16 (R)	–	> 8 (R)	4 (I)	2 (S)	2 (I)	<=32 (S)	≤20 (S)
13C	*Acinetobacter baumannii*	3	104–12-10	–	8 (S)	≤8 (S)	≤2 (S)	–	4 (S)	8 (S)	≤0.5 (S)	–	≤0.25 (S)	≤0.25 (S)	≤1 (S)	0.25 (S)	> 128 (R)	≤20 (S)
41C	*Acinetobacter baumannii*	2	104–12-10	–	32 (I)	16 (S)	4 (S)	–	8 (S)	8 (S)	1 (S)	–	≤0.25 (S)	0.5 (S)	≤1 (S)	≤0.25 (S)	> 128 (R)	≤20 (S)
45C	*Acinetobacter baumannii*	2	104–12-10	–	32 (I)	32 (I)	4 (S)	–	8 (S)	16 (I)	2 (S)	–	≤0.25 (S)	1 (S)	≤1 (S)	≤0.25 (S)	128 (I)	≤20 (S)
50E-B	*Acinetobacter baumannii*	2	104–12-10	–	8 (S)	≤8 (S)	≤2 (S)	–	2 (S)	4 (S)	≤0.5 (S)	–	≤0.25 (S)	≤0.25 (S)	≤1 (S)	≤0.25 (S)	128 (I)	≤20 (S)
51E	*Acinetobacter baumannii*	2	104–12-10	–	4 (S)	≤8 (S)	≤2 (S)	–	2 (S)	4 (S)	≤0.5 (S)	–	≤0.25 (S)	≤0.25 (S)	≤1 (S)	≤0.25 (S)	128 (I)	≤20 (S)
52E	*Acinetobacter baumannii*	2	104–12-10	–	8 (S)	≤8 (S)	≤2 (S)	–	4 (S)	8 (S)	≤0.5 (S)	–	≤0.25 (S)	≤0.25 (S)	≤1 (S)	≤0.25 (S)	128 (I)	≤20 (S)
12C-C	*Acinetobacter baumannii*	3	104–13-8	–	8 (S)	≤8 (S)	≤2 (S)	–	2 (S)	8 (S)	≤0.5 (S)	–	≤0.25 (S)	≤0.25 (S)	2 (S)	≤0.25 (S)	128 (I)	≤20 (S)
70C	*Acinetobacter nosocomialis*	2	2090-44-10	–	> 64 (R)	> 64 (R)	16 (I)	–	> 16 (R)	> 32 (R)	> 16 (R)	–	≤0.25 (S)	1 (S)	≤1 (S)	0.5 (S)	128 (I)	≤20 (S)
67C-B	*Acinetobacter* sp. *close to 13TU*	3	3045-12-10	–	64 (I)	32 (I)	≤2 (S)	–	> 16 (R)	> 32 (R)	2 (S)	–	≤0.25 (S)	0.5 (S)	2 (S)	1 (S)	128 (I)	≤20 (S)
59C-B	*Acinetobacter* sp. *close to 13TU*	3	3109-12-10	–	64 (I)	16 (S)	≤2 (S)	–	4 (S)	8 (S)	4 (S)	–	≤0.25 (S)	0.5 (S)	2 (S)	≤0.25 (S)	128 (I)	≤20 (S)
56C	*Acinetobacter ursingii*	2	4000-50-16	–	> 64 (R)	> 16 (R)	> 16 (R)	–	> 16 (R)	> 32 (R)	> 16 (R)	–	≤0.25 (S)	0.5 (S)	≤1 (S)	1 (S)	64 (S)	> 40 (R)
68E	*Acinetobacter baylyi*	2	–		4 (S)	8 (S)	≤2 (S)	–	4 (S)	8 (S)	≤0.5 (S)	–	≤0.25 (S)	≤0.25 (S)	≤1 (S)	≤0.25 (S)	≤32 (S)	≤20 (S)
25C	*Acinetobacter* sp.	2	4000-2-16	–	> 64 (R)	16 (S)	4 (S)	–	8 (S)	8 (S)	≤0.5 (S)	–	≤0.25 (S)	≤0.25 (S)	≤1 (S)	≤0.25 (S)	> 128 (R)	≤20 (S)
32E	*Acinetobacter* sp.	2	4000-2-16	–	16 (S)	≤8 (S)	≤2 (S)	–	16 (I)	8 (S)	≤0.5 (S)	–	≤0.25 (S)	≤0.25 (S)	≤1 (S)	≤0.25 (S)	128 (I)	≤20 (S)
65C	*Acinetobacter* sp.	3	4000-2-16	–	16 (S)	≤8 (S)	≤2 (S)	–	> 16 (R)	16 (I)	1 (S)	–	≤0.25 (S)	0.5 (S)	2 (S)	0.5 (S)	128 (I)	≤20 (S)
71C	*Acinetobacter* sp.	1	4064-4-1	–	8 (S)	≤8 (S)	≤2 (S)	–	8 (S)	8 (S)	1 (S)	–	≤0.25 (S)	≤0.25 (S)	2 (S)	≤0.25 (S)	≤32 (S)	≤20 (S)
73E	*Escherichia coli*	1	49–56-83	> 16 (R)	> 64 (R)	≤8 (S)	16 (I)	> 16 (R)	> 16 (R)	> 32 (R)	> 16 (R)	> 4 (R)	≤0.25 (S)	≤0.25 (S)	2 (S)	> 2 (R)	–	≤20 (S)
43E	*Escherichia coli*	2	49–58-19	> 16 (R)	> 64 (R)	≤8 (S)	4 (S)	> 16 (R)	8 (R)	16 (R)	16 (R)	> 4 (R)	≤0.25 (S)	≤0.25 (S)	4 (S)	> 2 (R)	–	≤20 (S)
95E	*Escherichia coli*	1	49–62-235	> 16 (R)	> 64 (R)	≤8 (S)	> 16 (R)	> 16 (R)	4 (S)	> 32 (R)	> 16 (R)	> 4 (R)	≤0.25 (S)	≤0.25 (S)	32 (I)	> 2 (R)	–	> 40 (R)
77E	*Escherichia coli*	1	49–122-83	> 16 (R)	> 64 (R)	≤8 (S)	8 (S)	> 16 (R)	4 (S)	32 (R)	> 16 (R)	> 4 (R)	≤0.25 (S)	≤0.25 (S)	4 (S)	> 2 (R)	–	≤20 (S)
7C	*Klebsiella pneumoniae*	3	–	> 16 (R)	16 (S)	≤8 (S)	4 (S)	1 (S)	≤0.5 (S)	≤1 (S)	≤0.5 (S)	≤2 (S)	0.5 (S)	≤0.25 (S)	≤1 (S)	≤0.25 (S)	> 128 (R)	≤20 (S)
87C	*Klebsiella pneumoniae*	2	–	> 16 (R)	4 (S)	≤8 (S)	4 (S)	1 (S)	≤0.5 (S)	≤1 (S)	≤0.5 (S)	≤2 (S)	≤0.25 (S)	≤0.25 (S)	2 (S)	≤0.25 (S)	≤32 (S)	≤20 (S)
12C-A	*Klebsiella aerogenes*	3	–	> 16 (R)	32 (I)	32 (I)	> 16 (R)	> 16 (R)	> 16 (R)	> 32 (R)	≤0.5 (S)	> 4 (R)	≤0.25 (S)	≤0.25 (S)	≤1 (S)	≤0.25 (S)	64 (S)	≤20 (S)
91C	*Raoultella planticola*	1	–	–	4 (S)	≤8 (S)	–	–	≤0.5 (S)	≤1 (S)	≤0.5 (S)	–	≤0.25 (S)	≤0.25 (S)	≤1 (S)	≤0.25 (S)	64 (S)	≤20 (S)
82E	*Enterobacter cloacae*	1	–	> 16 (R)	16 (S)	16 (S)	> 16 (R)	> 16 (R)	16 (R)	32 (R)	≤0.5 (S)	> 4 (R)	≤0.25 (S)	≤0.25 (S)	≤1 (S)	≤0.25 (S)	> 128 (R)	≤20 (S)
102C-B	*Serratia marcescens*	2	–	16 (I)	≤2 (S)	≤8 (S)	8 (S)	> 16 (R)	≤0.5 (S)	≤1 (S)	≤0.5 (S)	≤2 (S)	0.5 (S)	≤0.25 (S)	≤1 (S)	≤0.25 (S)	≤32 (S)	≤20 (S)
18C-A	*Morganella morganii*	3	–	> 16 (R)	4 (S)	≤8 (S)	> 16 (R)	> 16 (R)	2 (S)	≤1 (S)	≤0.5 (S)	> 4 (R)	4 (S)	≤0.25 (S)	8 (S)	≤0.25 (S)	> 128 (R)	≤20 (S)
101E	*Rahnella* sp.	1	–	–	4 (S)	≤8 (S)	–	–	≤0.5 (S)	≤1 (S)	≤0.5 (S)	–	0.5 (S)	≤0.25 (S)	≤1 (S)	≤0.25 (S)	≤32 (S)	≤20 (S)
71E	*Pseudononas aeruginosa*	1	207–1	–	> 64 (I)	> 64 (I)	–	–	> 16 (R)	> 32 (R)	> 16 (R)	–	> 8 (R)	> 8 (R)	> 32 (R)	> 2 (R)	> 128 (R)	> 40
18C-B	*Pseudononas aeruginosa*	3	108–2		4 (S)	4 (S)			2 (S)		≤0.5 (S)		0.5 (S)	0.5 (S)	4 (S)	≤0.25 (S)		> 40
96C	*Pseudononas aeruginosa*	1	641–0		≤2 (S)	≤2 (S)			1 (S)		2 (S)		1 (S)	≤0.25 (S)	4 (S)	0.5 (S)		40
89C	*Pseudononas aeruginosa*	1	696–16		4 (S)	4 (S)			1 (S)		≤0.5 (S)		1 (S)	0.5 (S)	2 (S)	≤0.25 (S)		> 40
59C-A	*Pseudononas fluorescens/putida*	3	–	–	8 (S)	≤8 (S)	–	–	2 (S)	16 (I)	1 (S)	–	1 (S)	2 (S)	≤1 (S)	≤0.25 (S)	> 128 (R)	> 40 (R)
67C-A	*Pseudononas fluorescens/putida*	3	–	–	8 (S)	≤8 (S)	–	–	1 (S)	16 (I)	≤0.5 (S)	–	1 (S)	1 (S)	≤1 (S)	≤0.25 (S)	> 128 (R)	> 40 (R)
82C	*Pseudononas fluorescens/putida*	1	–	–	16 (S)	16 (S)	–	–	2 (S)	32 (I)	≤0.5 (S)	–	0.5 (S)	4 (I)	≤1 (S)	≤0.25 (S)	> 128 (R)	> 40 (R)
66E	*Pseudomonas* sp.	3	–	–	16 (S)	16 (S)	–	–	4 (S)	16 (I)	≤0.5 (S)	–	2 (S)	2 (S)	2 (S)	≤0.25 (S)	> 128 (R)	> 40 (R)
67E-A	*Pseudomonas* sp.	3	–	–	4 (S)	≤8 (S)	–	–	1 (S)	16 (I)	≤0.5 (S)	–	1 (S)	2 (S)	≤1 (S)	≤0.25 (S)	> 128 (R)	> 40 (R)
102E	*Pseudononas* sp.	2	–	–	8 (S)	8 (S)	–	–	8 (S)	–	1 (S)	–	1 (S)	1 (S)	≤1 (S)	≤0.25 (S)	–	> 40 (R)
12E-A	*Stenotrophomonas maltophilia*	3	–	–	> 64 (R)	16 (S)	–	–	16 (I)	> 32 (R)	> 16 (R)	–	> 8 (R)	> 8 (R)	> 32 (R)	2 (I)	64 (S)	40 (S)
18E	*Stenotrophomonas maltophilia*	3	–	–	> 64 (R)	> 64 (R)	–	–	2 (S)	> 32 (R)	> 16 (R)	–	> 8 (R)	> 8 (R)	> 32 (R)	1 (S)	≤32 (S)	≤20 (S)
20C	*Stenotrophomonas maltophilia*	3	–	–	> 64 (R)	> 64 (R)	–	–	4 (S)	> 32 (R)	> 16 (R)	–	> 8 (R)	> 8 (R)	> 32 (R)	2 (I)	> 128 (R)	≤20 (S)
26E	*Stenotrophomonas maltophilia*	2	–	–	> 64 (R)	32 (I)	–	–	2 (S)	32 (I)	> 16 (R)	–	> 8 (R)	> 8 (R)	> 32 (R)	1 (S)	128 (I)	≤20 (S)
62C	*Stenotrophomonas maltophilia*	3	–	–	> 64 (R)	> 64 (R)	–	–	> 16 (R)	> 32 (R)	> 16 (R)	–	> 8 (R)	> 8 (R)	> 32 (R)	2 (I)	128 (I)	40 (S)
101C	*Stenotrophomonas maltophilia*	1	–	–	> 64 (R)	> 64 (R)	–	–	> 16 (R)	> 32 (R)	> 16 (R)	–	> 8 (R)	> 8 (R)	> 32 (R)	2 (I)	64 (S)	≤20 (S)
59E-C	*Chryseobacterium indologenes*	3	–	–	64 (I)	64 (I)	–	–	> 16 (R)	> 32 (R)	> 16 (R)	–	> 8 (R)	> 8 (R)	> 32 (R)	1 (S)	> 128 (R)	40 (I)
60E	*Chryseobacterium indologenes*	3	–	–	> 64 (R)	64 (I)	–	–	> 16 (R)	> 32 (R)	> 16 (R)	–	> 8 (R)	> 8 (R)	> 32 (R)	1 (S)	> 128 (R)	> 40 (R)
65E-B	*Chryseobacterium indologenes*	3	–	–	64 (I)	16 (S)	–	–	> 16 (R)	> 32 (R)	> 16 (R)	–	> 8 (R)	> 8 (R)	> 32 (R)	0.5 (S)	> 128 (R)	> 40 (R)
78E	*Chryseobacterium indologenes*	1	–	–	64 (I)	32 (I)	–	–	> 16 (R)	> 32 (R)	> 16 (R)	–	> 8 (R)	> 8 (R)	32 (I)	1 (S)	> 128 (R)	≤20 (S)
102C-A	*Chryseobacterium indologenes*	2	–	–	> 64 (R)	64 (I)	–	–	> 16 (R)	> 32 (R)	> 16 (R)	–	> 8 (R)	> 8 (R)	32 (I)	0.5 (S)	> 128 (R)	≤20 (S)
64E	*Aeromonas salmonicida*	3	–	–	≤2 (S)	≤8 (S)	–	–	≤0.5 (S)	2 (S)	≤0.5 (S)	–	≤0.25 (S)	≤0.25 (S)	≤1 (S)	≤0.25 (S)	≤32 (S)	≤20 (S)
78C	*Ochrobactrum* sp.	1	–	–	> 64 (R)	> 64 (R)	–	–	> 16 (R)	16 (I)	2 (S)	–	1 (S)	1 (S)	8 (S)	≤0.25 (S)	> 128 (R)	> 40 (R)

*POT* PCR-based ORF typing, *ABPC* Ampicillin, *PIPC* Piperacillin, *T/P* Tazobactam/piperacillin, *A/S*, Ampicillin/sulbactam, *CEZ* Cefazolin, *CAZ* Ceftazidime, *CTX* Cefotaxim, *CZOP* Cefozopran, *CPDX* Cefpodoxime, *IMP* Imipenem, *MEPM*, Meropenem, *AMK* Amikacin, *CPFX* Ciprofloxacin, *FOM* Fosfomycin, *ST* Sulfamethoxazole, *S* Susceptible, *I* Intermediate, *R* Resistant

**Table 3:** MLST, serotype, Fim type, and AMR genes of the isolates analysed by whole genome sequences

	*E. coli 43E*	*E. coli 73E*	*E. coli 77E*	*E. coli 95E*	*A. baumannii* 13C, 41C, 45C, 50E-B, 51E, 52E	*A. ursingii* 56C	*P. aeruginosa* 71E
MLST	ST131	ST131	ST131	ST131	ST130		ST235
Serotype	O25:H4	O25:H4	O25:H4	O25:H4			
*FimH* type	*fimH30*	*fimH30*	*fimH30*	*fimH30*			
Beta-lactam	*bla*_CTX-M-27_ (100%)	*bla*_CTX-M-27_ (100%)	*bla*_CTX-M-27_ (100%)	*bla*_CTX-M-14_ (100%) *bla*_TEM-1B_ (100%)	*bla*_ADC-166_ (97.74%) *bla*_OXA-430_ (99.88%)	*bla*_CARB-2_ (100%)	*bla*_PDC-35_ (100%) *bla*_OXA-50_ (99.87%) *bla*_IMP-1_ (100%) *bla*_TEM-1B_ (100%)
Aminoglycoside	*aph(3″)-Ib* (100%) *aph(6)-Id* (100%) *aadA5* (100%)	*aph(3″)-Ib* (100%) *aph(6)-Id* (100%)	*aph(3″)-Ib* (100%) *aph(6)-Id* (100%)	*aph(3″)-Ib* (100%) *aadA5* (100%) *aac(3)-IId* (99.88%) *aac(6′)-Ib3* (99.82%)		*aac(6′)-Ib3* (99.82%) *aph(3″)-Ib* (99.88%) *aph(3′)-Ia* (100%) *aph(6)-Id* (100%)	*aac(6′)-Iae* (100%) *aph(3′)-II* (100%) *aadA1* (100%)
Fluoroquinolone				*aac(6′)-Ib-cr* (99.49%)		*aac(6′)-Ib-cr* (99.42%)	
Fosfomycin							*fosA* (100%)
MLS (Macrolide, Lincosamide, Streptogramin B)	*mdf(A)* (97.81%) *mph(A)* (100%)	*mdf(A)* (97.81%)	*mdf(A)* (97.81%) *mph(A)* (100%)	*mph(A)* (100%) *mdf(A)* (97.81%)		*mrs(E)* (100%) *mph(E)* (100%)	
Phenicol				*cmlA1* (99.92%)		*floR* (98.35%)	*catB7* (100%)
Rifampicin						*ARR-3* (100%)	
Sulfonamide	*sul2* (100%) *sul1* (100%)	*sul2* (100%)	*sul2* (100%) *sul1* (100%)	*sul2* (100%) *sul1* (100%)		*sul2* (100%) *sul1* (100%)	*sul1* (100%)
Tetracycline	*tet(A)* (100%)	*tet(A)* (100%)	*tet(A)* (100%)			*tet(39)* (99.91%)	
Trimethoprim				*dfrA17* (100%)		*dfrA19* (100%)	

**Fig. 2:**
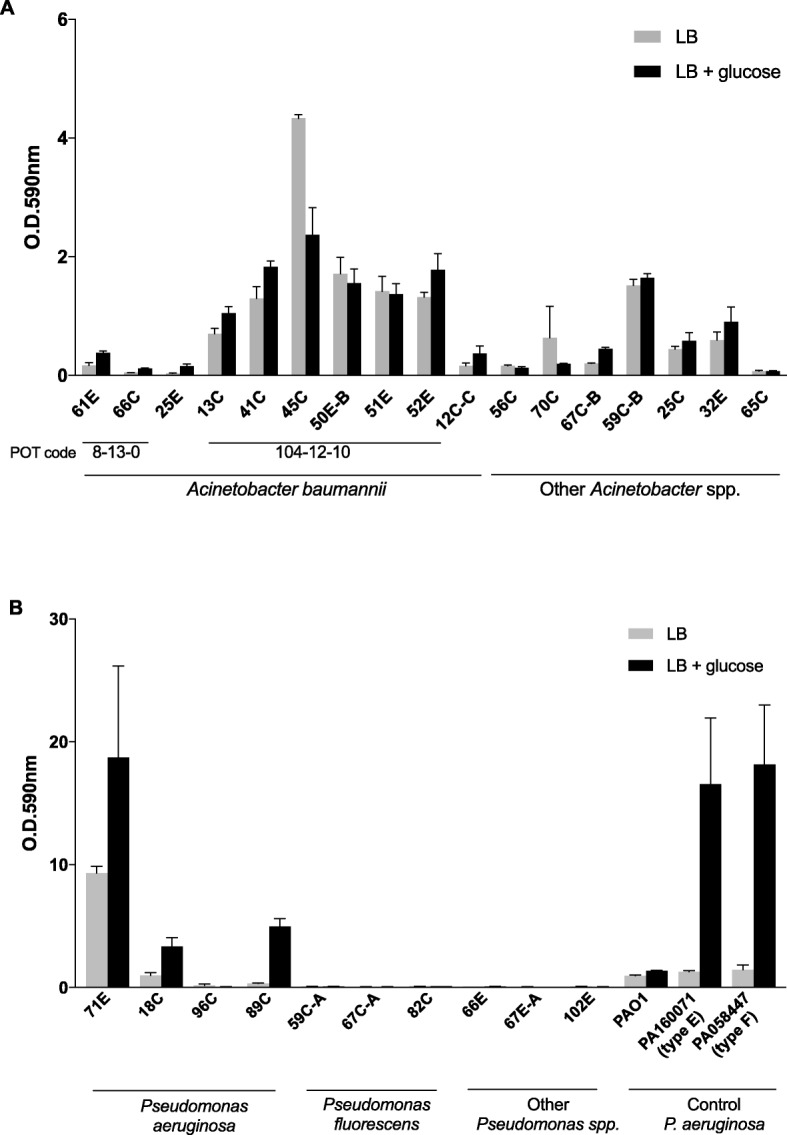
Biofilm production by Acinetobacter spp. isolates (**a**) and Pseudomonas spp. isolates (**b**). Bacteria were grown in LB medium in the absence or presence of 1% glucose. Biofilm formation was measured at OD_590nm_ using a microtiter plate biofilm-formation assay and the crystal violet dye. The average and standard error of the mean for each sample are shown

**Fig. 3:**
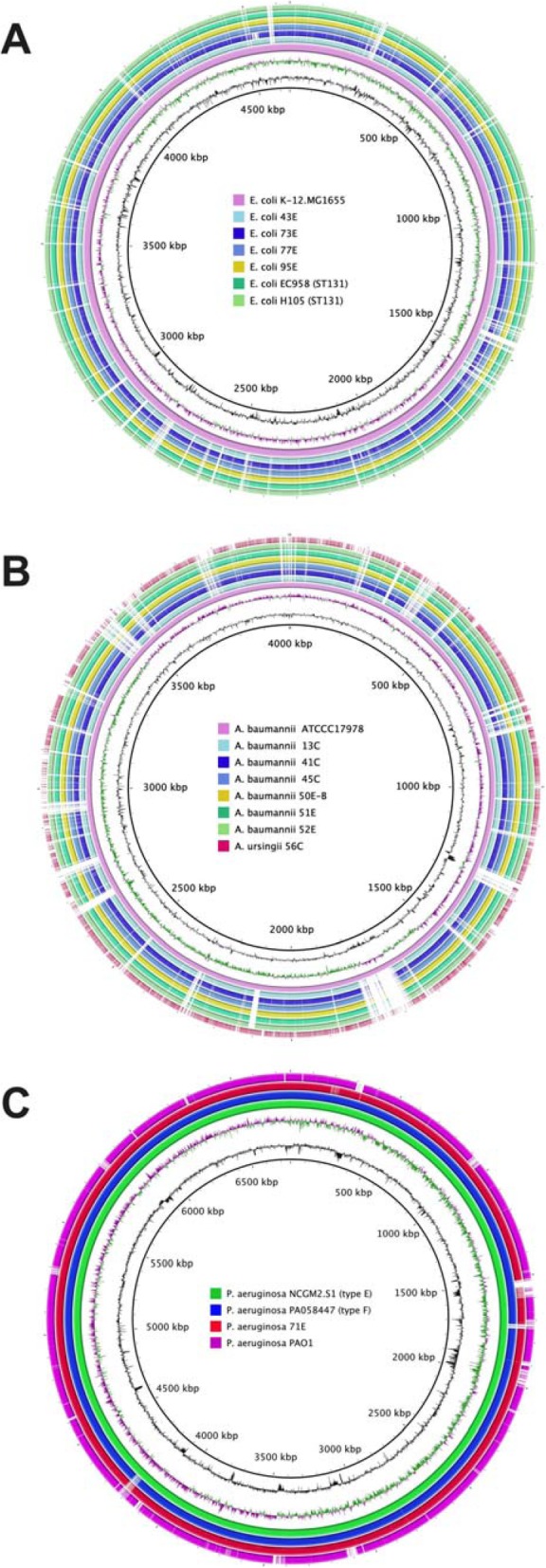
Comparison of genome sequences of *Escherichia coli* isolates (A), *Acinetobacter baumannii isolates* (B) and *Pseudomonas aeruginosa* isolates (C). (**a**) Comparison of genome sequences of four *E. coli* isolates. The unassembled sequences of *E. coli* isolates 43E, 73E, 77E, and 95E were aligned and compared to the complete genome sequence of *E. coli* K-12.MG1655 (accession number: NC_000913), *E. coli* EC958 (accession number: HG941718) and *E. coli* H105 (accession number: CP021454) strains. (**b**) Comparison of genome sequences of six *A. baumannii* isolates with the same POT code of 104–12-10 and *A. ursingii* isolate 56C. The unassembled sequences of these *A. baumannii* isolates were aligned and compared to the complete genome sequence of *A. baumannii* ATCC17987 strain (accession number: NZ_CP018664). (**c**) Comparison of genome sequences of *P. aeruginosa* NCGM2.S1 (type E), PA058447 (type F), 71E, and PAO1 strains. The unassembled sequences of *P. aeruginosa* isolate 71E and PA058447 strain were aligned and compared to the complete genome sequence of *P. aeruginosa* NCGM2.S1 (accession number: AP012280) and PAO1 (accession number: NC_002516) strains

Resistance of *A. baumannii* isolates to antibiotics varied among individual strains. To our surprise, the biofilm formation assay demonstrated that the six *A. baumannii* isolates (N^o^ 13C, 41C, 45C, 50E-B, 51E, and 52E) showed higher biofilm formation than the others (Fig. 2a). Remarkably, six isolates demonstrated the same POT code of 104–12-10, suggesting their similar origin. Interestingly, among six patients colonised with the same POT code, five patients were at the 2nd floor and one was at the 3rd floor (Table 2). Two patients carrying the isolates POT code 8–13-0 were at the same floor (3rd floor). MLST analysis using WGS data revealed the six *A. baumannii* belonged to ST130 (Table 3). Also, the genome comparison of these strains through BRIG using *A. baumannii* ATCC17978 strain as a reference exhibited high similarity among all the strains, with identical missing fragments in comparison with the ATCC17978 strain (Fig. 3b). Interestingly, among the nine isolates of the other *Acinetobacter* spp*.*, we found one (N^o^ 56C) displayed extensive resistance to multiple drugs (Table 2). Analysis of 16S rRNA and *rpoB* gene sequences identified this isolate as *A. ursingii* [23]. In addition, this strain was confirmed to carry extensive AMR genes against various groups of antimicrobials, which were not found in the other six *A. baumannii* isolates (Table 3).

Among the four *P. aeruginosa* isolates, N^o^ 71E exhibited the exclusive resistance to all tested ß-lactams, and to amikacin, ciprofloxacin, and fosfomycin as well. The *bla*_IMP-1_ gene was detected from isolate N^o^ 71E through PCR. Strikingly, the *P. aeruginosa* isolate N^o^ 71E exhibited outstanding biofilm formation which far surpassed any other *Pseudomonas* isolates and was equal to the ability of PA160071 and PA058447 strains in the presence of 1% glucose (Fig. 2b). Even when glucose was absent, isolate 71E revealed a stronger biofilm formation compared to PA160071 and PA058447 strains. WGS of isolate 71E indicated that this isolate belonged to the ST235 and carried type I integron with multiple resistance genes, ß-lactamases *bla*_IMP-1_, aminoglycoside-resistance genes *aac(6′)-Iae* and *aadA1*, and sulfamethoxazole-resistance gene *sul1* (Table 3). BLASTn comparison of WGS of isolate 71E, PA058447, NCGM2.S1, and PAO1strains using NCGM2.S1 as a reference, showed that isolate 71E showed similar characters to both NCGM2.S1 and PA058447 but distinct from PAO1 strain (Fig. 3c).

*C. indologenes* isolates showed broad resistance to all ß-lactams, including carbapenems but this resistance has been designated as intrinsic resistance by *bla*_IND_ group, the class B carbapenem-hydrolyzing ß-lactamase genes [24]. Similarly, *S. maltophilia* displayed extensive resistance to carbapenems and the other ß-lactams due to two intrinsic ß-lactamase genes, *bla*L1 (class B metallo-ß-lactamase (MBL)) and *bla*L2 (class A, functional group 2e, clavulanic acid susceptible cephalosporinase) [24]. Two *Klebsiella pneumoniae* isolates were resistant to ampicillin, which may be caused by their chromosomally-encoded *bla*_SHV_-related genes [25], but susceptible to the other ß-lactams. Other sporadic resistant isolates were also identified, such as *Klebsiella aerogenes*, *Enterobacter cloacae*, *Morganella morganii*, *Serratia marcescens*, and *Ochrobactrum* sp*.*, some of which displayed extensive resistance to ampicillins and cephalosporins due to their intrinsic resistance, for example, AmpC ß-lactamase CMY-108 in *K. aerogenes* [26], *E. cloacae* ß–lactamase A (IP, 8.8) and B (IP, 7.8) in *E. cloacae* [27], cephalosporinases encoded by *ampC-ampR* genes in *M. morganii* [28], and AmpC ß-lactamases in *S. marcescens* and *Ochrobactrum* sp. [24]. All of these isolates showed low biofilm formation ability.

## Discussion

ARB, as defined by growth on screening agar plates, most commonly *Acinetobacter, Enterobacteriaceae,* and *Pseudomonas* spp*.*, were detected in 37 of 98 (38%) LTCF residents. Moreover, some residents were colonised with more than one type of ARB. The presence of ARB in the oral niche poses a potential risk for pulmonary infections, in which certain kinds of Gram-negative bacteria such as *P. aeruginosa*, *E. coli*, *K. pneumonia* and the others have been reported [6]. Our findings reinforced the significant role of LTCF residents as reservoirs for ARB and also brings the attention to the colonisation by ARB in the oral environment besides the other well-documented body areas.

*E. coli* ST131 has been reported to be responsible for serious extraintestinal infections and medical implications [29]. Meanwhile, the rapid emergence and international dissemination of this clonal group has posed a critical threat to public health due to its significant AMR [30]. In our study, we also determined that all ESBL-producing *E. coli* isolates belonged to the most-virulent phylogroup B2, were *bla*_CTX-M-27_-producing O25:H4 ST131 *fimH30 E. coli*, and carried mutations in DNA sequences of the chromosomal QRDRs resulting in fluoroquinolone resistance. The mutations in the *gyrA* QRDR have been found to be the most frequent mutations among fluoroquinolone-resistant *E.coli* isolates [31]. The high virulence and resistance of *fimH30* lineage to fluoroquinolones, one of the most widely used antimicrobials for urinary tract infections, has made this lineage the most epidemiologically successful subclone of *E. coli* ST131 [32]. Considering the severe complications, MDR, and rapid propagation of this endemic clone, infection control and transmission prevention should be earnestly considered, especially within the vulnerable population in long-term care settings.

*Acinetobacter* has been recently responsible for multihospital outbreaks in temperate countries increasing the risk for residents in LTCFs, especially those receiving mechanical ventilation [33]. In our study there was no MDR *Acinetobacter* but seven out of 10 showed susceptibility to antimicrobials according to the MIC determination, six of which showed extensive biofilm production and showed a highly resistant phenotype on screening media. Besides the detection of ARB carrying multiple AMR genes, the presence of isolates with low MIC based on biochemical tests and no acquired AMR genes while still growing on CHROMagar™ ESBL or CHROMagar™ mSuperCARBA plates, and high biofilm-formation ability, was also a noteworthy finding. This inconsistence, in fact, reflects the difference in the AMR phenotype between a planktonic lifestyle of bacteria in MIC biochemical tests and biofilm lifestyle on agar plates, where bacteria are encased in an extracellular matrix that provides them tolerance and resistance mechanisms to combat antimicrobial challenges. For instance, the biofilm matrix provides a barrier for the penetration of antibiotics and hence, decreases bacteria susceptibility. Exopolysaccharides and extracellular DNA play a role in the resistance to antimicrobial agents, or secreted ß-lactamases in the matrix can degrade antimicrobials [34]. The growth of bacteria in the presence of an antimicrobial agent, despite their low MICs by Vitek-2 or the lack of AMR genes, may suggest their growability inside the human body with the presence of that antimicrobial agent. In other words, the low MIC of Vitek-2 does not necessarily mean the bacteria cannot grow in the presence of antimicrobials inside the human body, considering their biofilm lifestyle. From the viewpoint of hospital infection control, the mobile AMR genes or plasmids are significant concerns given their horizontal gene transfer from organism to organism within the health-care setting. However, from the viewpoint of clinical implications, both the AMR gene-carriers and biofilm-producers are important for their resistance phenotype inside the human body, either through the enzyme-mediated mechanism or the biofilm-based mechanism or through a combination of both. Moreover, our six isolates appeared to be clonally disseminated among the hospital ward. Peleg et al. emphasised that the attachment and biofilm formation ability of *A. baumannii* inside the body and on object surfaces took part in their virulence mechanisms and long endurance within health care settings, thus increased the potential for an epidemic outbreak [35]. Our results also support this hypothesis and lead to the conclusion that strong biofilm-producing *A. baumannii* should be regarded as a potential risk even though it lacks AMR genes.

*A. ursingii* 56C showed expanded resistance to various ß-lactams. *A. ursingii* is an uncommon opportunistic pathogen first described as a novel species in 2001 [36]. Sporadic cases involving serious bloodstream infections, in patients that are either immunocompromised or immunocompetent, have been reported [37, 38]. Although most of these isolates were susceptible to antimicrobial agents, the first report of a carbapenem-resistant *A. ursingii* from clinical isolates was described in Japan in 2010 [39] followed by a report of three carbapenemase-producing *A. ursingii* isolates in The Netherlands from September 2015 to June 2016 [40]. In our study, the *A. ursingii* isolate did not carry any carbapenemase gene such as *bla*_IMP-4_ and *bla*_OXA58_ but had some AMR genes as the ones carried by the isolates from The Netherland, for example, *bla*_CARB-2_, *aac(6′)Ib-cr*, *mph(E)*, *msr(E)*, *sul1*, and *tet(39)*.

Another leading emerging nosocomial pathogen that challenges the therapeutic treatment in clinical settings is *P. aeruginosa, which* confers both AMR phenotype and robust biofilm production, the so-called resistant biofilm-phenotype. Alternatively, Leibovitz et al. designated the biofilm covering the nasogastric-feeding tubes as reservoirs for biofilm-associated persistent *P. aeruginosa* in the oropharynges [41]. MBL genes coexist with other AMR elements and are commonly carried on mobile gene cassettes in class 1 or 3 integrons inserted into plasmids or in the chromosome [42], thus MBL-producing *P. aeruginosa* generally has a MDR phenotype. A 2005 outbreak of MDR *P. aeruginosa* carrying a novel chromosomally encoded class 1 integron, namely In113, later designated as type E integron by our group [8], has been reported in a neurosurgery ward in the Miyagi prefecture [9]. Complete genome sequence of a representative of this endemic cluster, NCGM2.S1 (previously named IMCJ2.S1) revealed that this strain harboured a *bla*_IMP-1_ gene cassette and a *aac(6′)-Iae* gene cassette in the integron In113, promoting the high level of MDR to ß-lactams and aminoglycosides, respectively, without the presence of any plasmids [10]. In the meantime, an epidemic clonal (designated as type F by our group) MDR *P. aeruginosa* in the Hiroshima region, represented by PA058447, was also noticed from a 9-year longitudinal molecular epidemiology study. This strain was proposed to have identical resistance element sequences as In113-carrying *P. aeruginosa* (type E) yet a disrupted *Intl1* by an IS*26* insertion and subsequent genomic reorganisation [11]. Interestingly, those two endemic clones were identified as having the same sequence type, ST235. Our detection of ST235 *P. aeruginosa* 71E with outstanding biofilm-formation ability, MDR phenotype, and close clonal origin with the epidemic ST235 MDR *P. aeruginosa* in Hiroshima region has called a conscientious attention.

Although *S. maltophilia* was not deeply investigated due to its natural resistance, the high prevalence of this species in this LTCF was also noteworthy since this pathogen has been recognised to produce nosocomial pneumonia in patients that are critically ill [43]. Inadequate empirical antimicrobial therapy over such high intrinsic resistance species may fail to defeat the infection, thus caution should be taken when administering medication empirically to those individuals.

Strokes, predominantly cerebral infarction and cerebral haemorrhage, were found to be a significant risk factor for oropharyngeal colonisation by ARB in LTCF. Gill et al. previously stated that stroke was significantly associated with disability in the elderly [44], requiring substantial assistance from care-givers with regards to dressing, toileting, eating, and other daily activities. In addition, faecal incontinence associated with diaper use and nursing aids have been recognised as risk factors for faecal carriage of ESBL-*Enterobacteriaceae* [45] and were likely linked to ARB transmission mediated via healthcare workers. This explanation is plausible as 14.5% of LTCF staff were colonised with ESBL-producers in the study of March et al. [7]. Besides, contaminated environments and mechanical equipment (e.g., nasogastric tubes, tracheostomy tubes, and urinary catheters) may accelerate the colonisation by nosocomial pathogens specifically those showing vigorous biofilm production. An outbreak of AMR *A. baumannii* due to the utilisation of contaminated tap water from hygiene sinks for oral care in an intensive care unit was reported [46]. The greater the advanced disability patients have, the more assistance and interaction with care-workers and medical-device operators are required, consequently facilitating the propagation of such microorganisms. In addition, patients who were fed with PEG tubes might undergo a reduction in mastication activity and salivary secretion, hence promoting the pathogenic colonisation inside the oral cavity. Since colonisation is an essential prerequisite for infection [45], the colonisation by ARB in LTCF raises a crucial challenge regarding the shortage of available efficient anti-infective agents once infection occurs.

Recently, to reduce the risk of “nursing home-acquired pneumonia” (NHAP), more effort have been taken to improve oral care such as implementing professional mouth care (brushing teeth, swabbing the mucosa, cleaning dentures, using mouthwash, having dental check-ups by professional dentists, or a combination of those methods) [47], being aware of dysphagia signs [48], improving the staff practices for oral care [49], or promoting oral hygiene, and swallowing ability of the LTCF residents [50]. Professional oral health care was proved to be cost-effective in declining the mortality and morbidity rates from NHAP in German healthcare settings [51]. In a systematic review by the Cochrane Library, despite the lack of high-quality evidence to determine the most effective measures to prevent NHAP, these studies proposed the efficacy in lowering pneumonia mortality through professional oral care when compared to usual oral care after a 24 month-follow up [47]. Whether the professional oral care will be an effective measure to reduce prevalence of ARB remains to be examined.

The study has several potential limitations that deserved to be discussed. First, as a limited number of patients participated in this surveillance, alternative possible risk factors might have not been identified. Second, no historical records of antibiotic use among the residents one year prior to this study were available, hindering the determination of the association between antimicrobial therapy and development of ARB. Third, this surveillance was conducted in a single LTCF, thus the overall prevalence of oral colonisation by ARB among other LTCFs in Japan remains to be elucidated. A similar study should be conducted in a large-scale long-term care system in order to comprehend the general situation and comparative evaluation.

## Conclusions

In summary, the present study detected high prevalence of AMR Gram-negative bacteria, ESBL-producing and carbapenem-resistant pathogens relevant to aspiration pneumonia, which carried the resistance genes on mobile elements such as plasmids or integrons or in the chromosome and/or are strong biofilm producers, in the oral cavity of LTCF residents. Health care workers involved in oral care should be aware of such ARB and pay special attention to transmission prevention and infection control to diminish the dissemination of ARB or the mobile resistance elements in LTCFs. Last but not least, with the rapid ageing of the Japanese society, surveillance initiatives and regional and national projects for infection control should not put aside the significant role of LTCFs or nursing homes in the healthcare network.

## Supplementary information

**Additional file 1: Table S1**. Primers used in present study. **Table S2**. Number of ARB, as defined by growth on screening media, isolated from oropharyngeal samples.

## Data Availability

All data generated or analysed during this study are included in this published article and its supplementary information files.

## Abbreviations

ARB: Antimicrobial-resistant bacteria
BRIG: Blast ring image generator
CI: Confidence interval
CLSI: By clinical laboratory standards institute
ESBL: Extended-spectrum ß-lactamase
LB: Luria-bertani
LTCF: Long-term care facility
MBL: Metallo-ß-lactamase
MDR: Multidrug resistant
MIC: Minimum inhibitory concentration
MLST: Multilocus sequence typing
NHAP: Nursing home-acquired pneumonia
OR: Odds ratios
PBS: Phosphate buffered saline
PEG: Percutaneous endoscopic gastrotomy
POT: PCR-based ORF typing
QRDR: Quinolone resistance-determining region
WGS: Whole genome sequences
