# Supplementary material for: Oral colonisation by antimicrobial-resistant Gram-negative bacteria among long-term care facility residents: prevalence, risk factors, and molecular epidemiology
Source: Antimicrob Resist Infect Control. 2020 Mar 4;9:45. doi: 10.1186/s13756-020-0705-1 (PMC7057508; doi:10.1186/s13756-020-0705-1)
Supplement: Supplementary file 1 — Additional file 1: Table S1. Primers used in present study. Table S2. Number of ARB, as defined by growth on screening media, isolated from oropharyngeal samples. [file 13756_2020_705_MOESM1_ESM.docx]

**Supplementary Table 1** Primers used in present study

| Primers | Sequence (5’ – 3’) | Product size (bp) | Reference |
| --- | --- | --- | --- |
| CTX-M-1-F | GCTGTTGTTAGGAAGTGTGC | 515 | Shibata et al. (2006) |
| CTX-M-1-R | CCATTGCCCGAGGTGAAG |  |  |
| CTX-M-2-F | ACGCTACCCCTGCTATTT | 780 | Shibata et al. (2006) |
| CTX-M-2-R | GCTTTCCGCCTTCTGCTC |  |  |
| CTX-M-8-F | CGGATGATGCTAATGACAAC | 569 | Shibata et al. (2006) |
| CTX-M-8-R | GTCAGATTGCGAAGCGTC |  |  |
| CTX-M-9-F | GCAGATAATACGCAGGTG | 393 | Shibata et al. (2006) |
| CTX-M-9-R | CGGCGTGGTGGTGTCTCT |  |  |
| TEM-F | CCGTGTCGCCCTTATTCC | 824 | Yagi et al. (2000) |
| TEM-R | AGGCACCTATCTCAGCGA |  |  |
| SHV-F | TCGGGCCGCGTAGGCATGAT | 625 | Leung et al. (1997) |
| SHV-R | AGCAGGGCGACAATCCCGCG |  |  |
| IMP-F | GGAATAGAGTGGCTTAAYTCTC | 232 | Poirel et al. (2011) |
| IMP-R | GGTTTAAYAAAACAACCACC |  |  |
| IMP-1-F | CTACCGCAGCAGAGTCTTTG | 587 | Kouda et al. (2009) |
| IMP-1-R | AACCAGTTTTGCCTTACCAT |  |  |
| OXA-F | GCGTGGTTAAGGATGAACAC | 438 | Poirel et al. (2011) |
| OXA-R | CATCAAGTTCAACCCAACCG |  |  |
| NDM-F | GGTTTGGCGATCTGGTTTTC | 621 | Poirel et al. (2011) |
| NDM-R | CGGAATGGCTCATCACGATC |  |  |
| KPC-F | CGTCTAGTTCTGCTGTCTTG | 798 | Poirel et al. (2011) |
| KPC-R | CTTGTCATCCTTGTTAGGCG |  |  |
| VIM-F | GATGGTGTTTGGTCGCATA | 390 | Poirel et al. (2011) |
| VIM-R | CGAATGCGCAGCACCAG |  |  |
| VIM-2-F | ATGTTCAAACTTTTGAG | 801 | Kouda et al. (2009) |
| VIM-2-R | CTACTCAACGACTGAGCG |  |  |
| FIM-1-F | GAAGCACATGGAAAACTGGG | 432 | Pollini et al. (2013) |
| FIM-1-R | GATGGGCGAATGAGACAGC |  |  |

**Supplementary Table 2** Number of ARB, as defined by growth on screening media, isolated from oropharyngeal samples.

| **Microorganisms** | **Number of isolates; % colonised** | | |
| --- | --- | --- | --- |
|  | Total, *n=98* | Men, *n=29* | Women, *n=69* |
| *Acinetobacter baumannii* | 10 (10%) | 2 (7%) | 8 (12%) |
| Other *Acinetobacter* sp. | 9 (9%) | 1 (3%) | 8 (12%) |
| *Stenotrophomonas maltophilia* | 6 (6%) | 2 (7%) | 4 (6%) |
| *Chryseobacterium indologenes* | 5 (5%) | 2 (7%) | 3 (4%) |
| *Escherichia coli* | 4 (4%) | 3 (10%) | 1 (1%) |
| *Pseudomonas aeruginosa* | 4 (4%) | 3 (10%) | 1 (1%) |
| *Pseudomonas fluorescens/putida* | 3 (3%) |  | 3 (4%) |
| Other *Pseudomonas* sp. | 3 (3%) | 1 (3%) | 2 (3%) |
| *Klebsiella pneumoniae* | 2 (2%) | 1 (3%) | 1 (1%) |
| *Klebsiella aerogenes* | 1 (1%) |  | 1 (1%) |
| *Raoultella planticola* | 1 (1%) | 1 (3%) |  |
| *Enterobacter cloacae* | 1 (1%) |  | 1 (1%) |
| *Serratia marcescens* | 1 (1%) | 1 (3%) |  |
| *Morganella morganii* | 1 (1%) | 1 (3%) |  |
| *Rhanella* sp. | 1 (1%) |  | 1 (1%) |
| *Aeromonas salmonicida* | 1 (1%) | 1 (3%) |  |
| *Ochrobactrum* sp. | 1 (1%) |  | 1 (1%) |
